# Supplementary material for: Identification of photoperiod-induced specific miRNAs in the adrenal glands of Sunite sheep (Ovis aries)
Source: Front Vet Sci. 2022 Jul 22;9:888207. doi: 10.3389/fvets.2022.888207 (PMC9354845; doi:10.3389/fvets.2022.888207)
Supplement: Supplementary file 1 [file Data_Sheet_1.ZIP › Supplementary file/miRDeep2 file.zip!/miRDeep2 file/Know_Structure/SPLP42A3/oar-mir-541.pdf]

miRBase precursor : oar-mir-541
Total read count : 104
oar-miR-541-5p read count : 94
oar-miR-541-3p read count : 10
remaining reads : 0

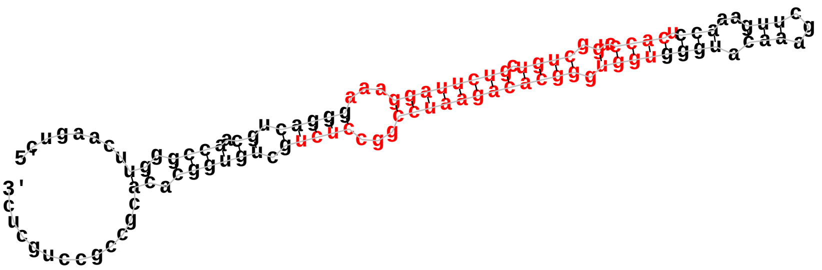

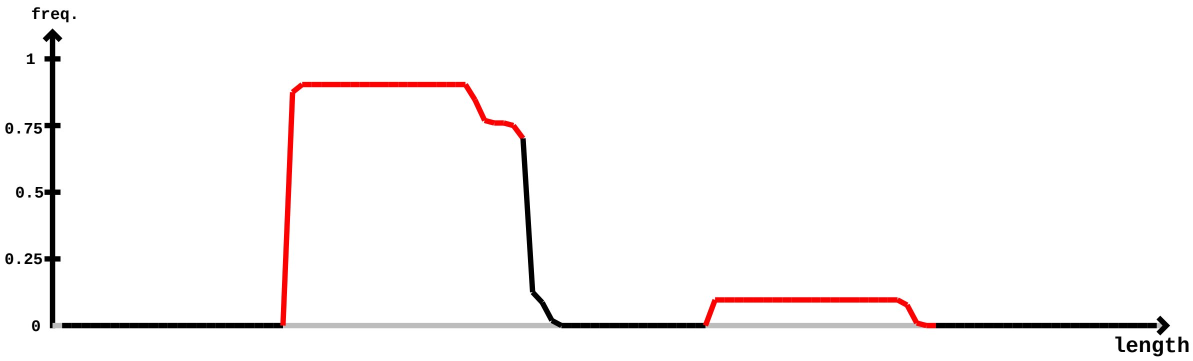

oar-miR-541-3p

oar-miR-541-5p

5'- cugaacuugggccaacgucagggaaaggauucugcugucggucccacuccaaaguucgaaacauggguggugggcacagaauccggccucugcuguggcacacgccgccugcuc -3'	exp		
........((((..(((.(((((...((((((((.((((.(..((((.(((..(((...))).)))))))).))))))))))))...))))).)))(((....)))))))....	reads	mm	sample
.......................aaaggauucugcugucggu..	5	0	seq
.......................aaaggauucugcugucgguc...	8	0	seq
.......................aaaggauucugcugucggucc..	1	0	seq
.......................aaaggauucugcugucgguccca..	1	0	seq
.......................aaaggauucugcugucggucccac...	5	0	seq
.......................aaaggauucugcugucggucccacu..	58	0	seq
.......................aaaggauucugcugucggucccacuc...	4	0	seq
.......................aaaggauucugcugucggucccacucc..	7	0	seq
.......................aaaggauucugcugucggucccacucca...	2	0	seq
........................aaggauucugcugucggu..	1	0	seq
........................aaggauucugcugucggucccacu..	2	0	seq
....uggugggcacagaauccggc...........................	2	0	seq
....uggugggcacagaauccggcc..........................	7	0	seq
....uggugggcacagaauccggccu.........................	1	0	seq
